# Chronic Respiratory Disorders and Their Treatment among Older People with Intellectual Disability and/or Autism Spectrum Disorder in Comparison with the General Population

**DOI:** 10.3390/healthcare5030040

**Published:** 2017-08-01

**Authors:** Anna Axmon, Peter Höglund, Gerd Ahlström

**Affiliations:** 1 Division of Occupational and Environmental Medicine, Lund University, SE-221 00 Lund, Sweden; anna.axmon@med.lu.se; 2 Division of Clinical Chemistry and Pharmacology, Lund University, SE-221 00 Lund, Sweden; peter.hoglund@med.lu.se; 3 Department of Health Sciences, Lund University, SE-221 00 Lund, Sweden

**Keywords:** asthma, pulmonary disease, chronic obstructive, middle aged, aged, diagnosis, delivery of health care, pharmaceutical preparations

## Abstract

Respiratory disorders are common among people with intellectual disabilities (ID). However, few studies have investigated these disorders among older people with ID. We identified 7936 people, aged 55+ years, with ID and a reference cohort from the general population. Data on diagnoses of chronic respiratory disorders, with a focus on asthma and chronic obstructive pulmonary disease (COPD), were collected, as was information on health care visits due to such disorders. We also added data on the prescription of drugs for obstructive airway diseases. Whereas the risk of having at least one diagnosis of asthma during the study period was similar in the two cohorts, people with ID were less likely than the general population to have been diagnosed with COPD. The same was found for health care visits due to asthma and COPD, respectively. The patterns of drug prescription were similar among people with ID and the general population, with the exception of adrenergics for systemic use, which were more commonly prescribed to people with ID. Thus, older people with ID do not seem to have an increased risk of asthma or COPD. Moreover, the indications are that when diagnosed with any of these disorders, they receive treatment adapted to their particular needs.

## 1. Introduction

Respiratory disorders are more common among people with intellectual disabilities (ID) than in the general population [1]. Also, people with ID are more likely to visit the emergency department for respiratory diseases [2], and hospital admissions are more frequent [3,4], of longer duration, and have a higher likelihood of recurring among people with ID than in the general population [3]. Compared to the general population, people with ID are more likely to die from respiratory infections [5,6]. Indeed, respiratory diseases are one of the most common causes of death among people with ID [7,8,9,10,11,12].

Asthma and chronic obstructive pulmonary disease (COPD) are two types of chronic inflammatory respiratory diseases. Several [13,14,15,16,17,18] but not all [1] studies indicate that asthma is more common among people with ID than in the general population. However, the increased risk is not as evident among people aged 55 years or more [18]. Nevertheless, asthma is more likely to cause death among people with ID than in the general population [19], and people with ID and asthma are more likely to be hospitalized than people with asthma without ID [20]. COPD in people with ID has been less studied. However, indications are that it is less common in populations with ID than in the general population [6,17,18,21], regardless of age [18].

Both asthma [22,23] and COPD [24,25] have been found to be associated with a high comorbidity burden. Also, people with uncontrolled (i.e., untreated) asthma have a lower quality of life than those with partially or fully controlled asthma [26], and a higher severity of COPD has been found to be associated with lower health-related quality of life (HRQoL), measured by the generic EuroQoL-5D (EQ-5D) [27] and the disease specific instrument COPD assessment test (CAT) [28]. However, comorbidities and demographics (sex and age) as well as socioeconomic factors influenced the HRQoL [27,28]. Therefore, it is of importance that these people receive comprehensive medical examinations and are treated properly according to their individual needs. Among people with ID, who are already disadvantaged when it comes to comorbidity [1,29] and quality of life [30], it is even more important.

Asthma and COPD are commonly treated using two drug components; a long-term control medication to reduce the number of attacks and rescue medications for symptom relief during attacks. Both long-term and rescue medication may be provided either as an inhaler or for oral intake. Studies regarding asthma treatment among people with ID have found that 86% are prescribed inhaled medications [31]. However, due to their cognitive impairment, they are likely to require support with the use of an inhaler [32]. This is especially important considering recent findings about health behavior risk factors for death due to asthma in a community based case-control study. People in the general population with poor inhaler technique were found to have a somewhat increased risk of asthma death [19].

Allergies and asthma affect individuals in all age groups but are often unrecognized or undertreated in the elderly [33]. Even so, aging increases the prevalence of asthma [22] and COPD [34] in the general population. Also among people with ID, the frequency of respiratory conditions has been found to increase with age [35], yet only a limited number of studies have focused on the prevalence and treatment of asthma and/or COPD among older people with ID.

The aim of the present study was to describe diagnoses and treatment of asthma and COPD among older people with ID in relation to their age peers in the general population and, furthermore, to relate diagnoses to treatment in these two populations.

## 2. Materials and Methods

This study is register based, using Swedish national registers both to establish the study cohorts and to identify outcomes (diagnoses and drug prescriptions). The two study cohorts have been described in detail elsewhere [1,29,36,37]. Briefly, all people aged 55+ years and alive at the end of 2012, and having received at least one measure of service or support provided for people with ID and/or autism spectrum disorder (ASD) during 2012, comprised the ID cohort (*n* = 7936, 3609 women and 4327 men). As we were not able to separate those with ID with or without ASD from those with ASD only, we used having received support as a proxy for ID. A referent cohort (gPop cohort) was randomly selected from the general population using a one-to-one match by sex and year of birth.

Data on diagnoses made in inpatient and outpatient specialist care during the study period (2006–2012) were collected from the Swedish National Patient Register. Both primary and secondary diagnoses were used to determine if a person had a diagnosis of a chronic respiratory disease. Although the main focus was on asthma (J45 in the International Statistical Classification of Diseases and Related Health Problems 10th Revision (ICD-10)) and other chronic obstructive pulmonary diseases (COPD; J44), we also present data on diagnoses of other chronic respiratory diseases, i.e., bronchitis, not specified as acute or chronic (J40), simple and mucopurulent chronic bronchitis (J41), unspecified chronic bronchitis (J42), emphysema (J43), status asthmaticus (J46), and bronchiectasis (J47).

Treatment for respiratory disorders was assessed using the prescription of drugs as well as health care utilization estimated by inpatient and outpatient specialist care due to (i.e., with primary diagnosis) any of the disorders listed above. The Swedish National Register of Prescribed Drugs was used to collect information on dispensed drugs for obstructive airway diseases (R03 in the Anatomical Therapeutic Chemical (ATC) classification system), which were analyzed separately for adrenergics and inhalants (R03A), glucocorticoids (R03BA), anticholinergics (R03BB), adrenergics for systemic use (R03C), and other systemic drugs (R03D).

Relative risks (RRs) with 95% confidence intervals (CIs) were estimated using Generalized Linear Models (GENLIN) in IBM SPSS Statistics 23.0. The results are only presented for groups with five or more observations. P-values below 0.05 are considered statistically significant.

Approval for the study was obtained from the Regional Ethical Review Board in Lund (no. 2013/15). The National Board of Health and Welfare performed a separate secrecy review in 2014 before providing access to the data. All analyses were performed using anonymized datasets.

## 3. Results

### 3.1. Health Care Utilization

People with ID were less likely than those in the gPop cohort to have had at least one inpatient or outpatient specialist visit due to chronic respiratory diseases (Table 1). The results were similar for men and women. There were no differences between men and women in either cohort. The pattern was the same for visits due to COPD. There was no statistically significant difference between the two cohorts with respect to health care utilization for asthma, although, when stratifying on sex, the men in the ID cohort had borderline significantly lower health care utilization than the men in the gPop cohort. Also, within the ID cohort, men were less likely than women to have had an inpatient or outpatient specialist visit due to asthma. This effect of sex was not found in the gPop cohort.

### 3.2. Drug Prescriptions

People in the ID cohort were less likely than people in the gPop cohort to be prescribed at least one drug for an obstructive airway disease (ATC-code R03; 12% vs. 19%; RR 0.64 [0.59–0.69]). The same pattern was found in separate analyses of men (10% vs. 16%; RR 0.64 [0.58–0.72]) and women (14% vs. 22%; RR 0.63 [0.57–0.70]). Within both the ID (RR 0.74 [0.65–0.83]) and the gPop cohort (RR 0.72 [0.66–0.79]), men were less likely than women to be prescribed at least one drug for an obstructive airway disease. All drugs for obstructive airway diseases that were prescribed and dispensed during the study period are described in Table 2.

### 3.3. Diagnoses

People in the ID cohort were less likely than those in the gPop cohort to have at least one diagnosis (including both primary and secondary diagnoses) of chronic respiratory disease during the study period (Table 3). The effect was slightly larger among men than among women. Within the ID but not the gPop cohort, men were less likely than women to have at least one diagnosis of chronic respiratory disease during the study period.

The most common diagnoses in both the ID and the gPop cohort were asthma and COPD (Table 3).

Among those with at least one diagnosis of asthma or COPD, COPD was the most common diagnosis in the ID cohort and asthma in the gPop cohort (Table 3).

Whereas the risk of diagnosis of asthma was similar in the two cohorts, diagnoses of COPD were less common in the ID cohort than in the gPop cohort (Table 3). The patterns were similar in separate analyses for women and men. Men were less likely to be diagnosed with asthma in both cohorts. There were, however, no sex differences with respect to the diagnosis of COPD in either cohort.

### 3.4. Diagnoses vs. Drug Prescriptions

In the ID cohort, 698 people had at least one prescription of drugs for an obstructive airway disease but no diagnosis of asthma or COPD during the study period (Table 4). A further 26 had at least one diagnosis of asthma and/or COPD but no prescription of drugs for an obstructive airway disease. In the gPop cohort, 1122 people had a prescription but no diagnosis, and 28 had a diagnosis but no prescription.

**Figure 1:**
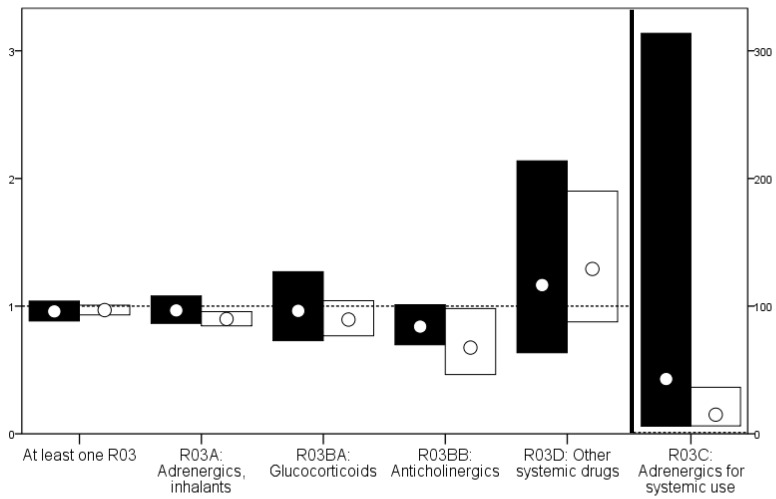
Relative risks with 95% confidence intervals for the prescription of drugs among people with COPD (black) and asthma (white).

Among those with a diagnosis of COPD during the study period, people in the ID cohort were more likely than those in the gPop cohort to be prescribed adrenergics for systemic use (Figure 1).

The results were similar when stratified by sex, although the number of men with prescriptions of adrenergics for systemic use was too low to investigate (Table 5). Among those with a diagnosis of asthma, people in the ID cohort were less likely than those in the gPop cohort to be prescribed adrenergics, inhalants, and anticholinergics but more likely to be prescribed adrenergics for systemic use (Table 5) No differences were found for the other drug types. When stratifying by sex, the differences were primarily found among the women (Table 5).

## 4. Discussion

Whereas the risk of having been diagnosed with asthma was the same for people with ID as for the general population, diagnosis of COPD was less common among people with ID. Among those with asthma or COPD, people with ID were more likely than those in the general population to be prescribed adrenergics for systemic use.

We used the National Patient Register to collect information on respiratory disorders. A strength of this is that the register has more or less complete coverage of inpatient and outpatient specialist care in Sweden. A limitation is that it does not include visits to primary care. As both asthma and COPD are commonly treated in primary care, we are likely to have failed to include all cases of these diseases. This is reflected in the high number of people without diagnoses but with prescriptions of at least one drug for obstructive airway diseases. People who seek inpatient or outpatient specialist care for their asthma/COPD may be suspected to be more severe cases than those who do not. Thus, people with severe asthma/COPD are likely to be overrepresented in both the ID and gPop cohort, whereas those with mild asthma/COPD may be underrepresented or even absent. The numbers in the present study should therefore not be used to estimate prevalence but only for comparisons between people with ID and the general population. This is also supported by the lower percentage of people with asthma found in the gPop cohort than in other studies on middle-aged and older people [38,39,40]. Another potential problem with using data from secondary care only is that differences in access to such care would appear as differences in disease prevalence. This is further discussed below.

So far, studies presenting results on the risk of asthma among people with ID are somewhat contradictory, with some finding an increased risk [13,15,16,17,18] and others not [41,42,43]. Possible explanations for this may include differences in age groups, geographical area, and the time period studied. Moreover, the type of data used varies, with some using primary care data [15,16,17,18,43] and others using different types of survey data [13,41,42]. The most obvious explanation for the lack of a difference between the ID cohort and the gPop cohort regarding asthma diagnoses is that the two cohorts do not differ in asthma prevalence, which is supported by a previous study presenting a decreased difference in the prevalence of asthma between people with ID and the general population in age groups older than 55 years [18]. This could be the effect of a survival mechanism, i.e., that people with ID and asthma tend to die at an earlier age. The lack of difference between the two cohorts could also arise from differences in health care utilization. For example, if people with ID have an increased risk of asthma but tend to visit primary rather than secondary care and the opposite is true for the general population, the data from inpatient and outpatient specialist care would indicate similarities in asthma prevalence. However, as diagnosing asthma may be difficult in people with ID, it may be argued that people with ID and asthmatic symptoms are more likely to visit and be diagnosed in specialist care than in primary care.

The decreased risk of COPD among people with ID found in the present study is in line with previous research [6,16,17,18,21], despite the differences in age groups studied. Tobacco smoke is the most important cause of COPD [44], and people with ID are less likely to smoke than the general population [45,46,47]. Thus, differences in smoking habits between people with ID and the general population is a plausible link between ID and a decreased risk of COPD. The selection of healthier people with ID into the older age groups, as discussed for asthma above, is not evident for COPD.

Asthma is classified as an ambulatory care sensitive (ACS) condition [48]. It has been suggested that rates of hospitalization for ACS conditions may be used as a measure of access to and the quality of primary care [49]. Based on this, it has been suggested that primary health care for asthma for people with ID is suboptimal compared with the general population [50]. In the present study, we investigated asthma-related inpatient and outpatient specialist visits rather than hospitalization. Doing this, we found no differences between people with ID and the general population, with the possible exception of a smaller number with visits among men with ID. Thus, the Swedish health care system seems to be able to properly care for older people with ID and asthma.

Among those with asthma and/or COPD, people with ID were more likely than the general population to be prescribed adrenergics for systemic use. We believe that the main explanation for this is that these are easier to administer than drugs provided in inhalers, especially when it comes to people with more severe ID.

## 5. Conclusions

Both asthma and COPD are chronic conditions that may have a large impact on a person’s quality of life, everyday living, and long term health, especially when insufficiently treated. Among older people in general and older people with ID in particular, it is important to ensure not only that treatment is provided in proper amounts, but also that it is administered in a way that is suitable for a patient with limited abilities. Indications are that care provided to asthma/COPD patients with ID indeed is adapted to the particular needs of these patients.

## Figures and Tables

**Table 1:** The number of people with at least one inpatient or outpatient specialist visit due to different types of chronic respiratory diseases in a cohort of people with intellectual disabilities (ID, *n* = 7936) and a same-sized referent sample from the general population (gPop), matched by sex and year of birth.

Primary Diagnosis at Visit	gPop				ID						
	All	Women	Men	Women vs. Men	All		Women		Men		Women vs. Men
	*n* (%)	*n* (%)	*n* (%)	RR ^b^ (95% CI)	*n* (%)	RR ^a^ (95% CI)	*n* (%)	RR ^a^ (95% CI)	*n* (%)	RR ^a^ (95% CI)	RR ^b^ (95% CI)
Any	252 (3)	119 (3)	133 (3)	0.93 (0.73–1.19)	152 (2)	0.60 (0.49–0.74)	77 (2)	0.65 (0.49–0.96)	75 (2)	0.56 (0.43–0.75)	0.81 (0.59–1.11)
Bronchitis, not specified as acute or chronic	15 (0)	7 (0)	8 (0)	0.95 (0.35–2.63)	14 (0)	0.93 (0.45–1.93)	6 (0)	0.86 (0.29–2.55)	8 (0)	1.00 (0.38–2.66)	1.11 (0.39–3.20)
Simple and mucopurulent chronic bronchitis	13 (0)	5 (0)	8 (0)	1.34 (0.44–4.08)	<5		<5		<5		
Unspecified chronic bronchitis	10 (0)	8 (0)	<5		6 (0)	0.60 (0.22–1.65)	5 (0)	0.63 (0.21–1.91)	<5		
Emphysema	10 (0)	<5	6 (0)		<5		<5		<5		
Other chronic obstructive pulmonary disease (COPD)	137 (2)	60 (2)	77 (2)	1.07 (0.77–1.50)	64 (1)	0.47 (0.35–0.63)	26 (1)	0.43 (0.27–0.69)	38 (1)	0.49 (0.34–0.73)	1.22 (0.74–2.00)
Asthma	105 (1)	51 (1)	54 (1)	0.88 (0.60–1.29)	85 (1)	0.81 (0.61–1.08)	49 (1)	0.96 (0.65–1.42)	36 (1)	0.67 (0.44–1.01)	0.61 (0.40–0.94)
Status asthmaticus	10 (0)	<5	6 (0)		6 (0)	0.60 (0.22–1.65)	<5		<5		
Bronchiectasis	9 (0)	6 (0)	<5		<5		<5		<5		

^a^: ID vs. gPop; ^b^: Within the ID cohort. Note: Relative risks (RRs) are given with 95% confidence intervals (CIs). RRs are presented only if both groups to be compared are comprised of at least five individuals.

**Table 2:** The number of people with at least one prescription of drugs for obstructive airway diseases (ATC-code R03) in a cohort of people with intellectual disabilities (ID) and a referent sample from the general population (gPop).

Drug	gPop			ID		
	Total	Women	Men	Total	Women	Men
R03AC02: salbutamol	299	180	119	255	143	112
R03AC03: terbutaline	758	421	337	379	218	161
R03AC12: salmeterol	43	21	22	13	9	4
R03AC13: formoterol	186	102	84	74	40	34
R03AC18: indacaterol	13	6	7	1	0	1
R03AK06: salmeterol and fluticasone	141	79	62	86	52	34
R03AK07: formoterol and budesonide	472	235	237	203	108	95
R03AK08: formoterol and beclometasone	5	3	2	15	9	6
R03AL02: salbutamol and ipratropium bromide	32	14	18	77	35	42
R03BA01: beclometasone	23	12	11	21	6	15
R03BA02: budesonide	633	353	280	314	179	135
R03BA05: fluticasone	43	23	20	69	39	30
R03BA07: mometasone	26	14	12	6	4	2
R03BA08: ciclesonide	1	0	1	0	0	0
R03BB01: ipratropium bromide	103	56	47	73	30	43
R03BB04: tiotropium bromide	260	137	123	109	52	57
R03BC01: cromoglicic acid	11	8	3	0	0	0
R03CA02: ephedrine	2	1	1	0	0	0
R03CC02: salbutamol	9	6	3	76	44	32
R03CC03: terbutaline	14	7	7	176	93	83
R03CC12: bambuterol	2	1	1	49	28	21
R03DA02: choline theophyllinate	2	0	2	52	26	26
R03DA04: theophylline	5	3	2	28	12	16
R03DC03: montelukast	86	44	42	61	32	29
R03DX07: roflumilast	5	0	5	2	1	1

**Table 3:** The number of people with at least one diagnosis (primary or secondary) of different types of chronic respiratory diseases in a cohort of people with intellectual disabilities (ID, *n* = 7936, 3609 women and 4327 men) and a same-sized referent sample from the general population (gPop), matched by sex and year of birth.

Diagnosis	gPop				ID						
	All	Women	Men	Women vs. Men	All		Women		Men		Women vs. Men
	*n* (%)	*n* (%)	*n* (%)	RR ^b^ (95% CI)	*n* (%)	RR ^a^ (95% CI)	*n* (%)	RR ^a^ (95% CI)	*n* (%)	RR ^a^ (95% CI)	RR ^b^ (95% CI)
Any	434 (5)	205 (6)	229 (5)	0.93 (0.78–1.12)	302 (4)	0.70 (0.60–0.80)	158 (4)	0.77 (0.63–0.94)	144 (3)	0.63 (0.51–0.77)	0.76 (0.61–0.95)
Bronchitis, not specified as acute or chronic	19 (0)	7 (0)	12 (0)	1.43 (0.56–3.63)	21 (0)	1.11 (0.60–2.05)	8 (0)	1.14 (0.42–3.15)	13 (0)	1.08 (0.50–2.37)	1.34 (0.56–3.33)
Simple and mucopurulent chronic bronchitis	21 (0)	7 (0)	14 (0)	1.69 (0.67–4.13)	<5		<5		<5		
Unspecified chronic bronchitis	21 (0)	13 (0)	8 (0)	0.51 (0.21–1.24)	11 (0)	0.54 (0.25–1.09)	8 (0)	0.62 (0.26–1.48)	<5		
Emphysema	19 (0)	7 (0)	12 (0)	1.43 (0.56–3.63)	5 (0)	0.26 (0.10–0.70)	<5		<5		
Other chronic obstructive pulmonary disease (COPD)	233 (3)	104 (3)	129 (3)	1.04 (0.80–1.33)	125 (2)	0.54 (0.43–0.67)	53 (1)	0.51 (0.37–0.71)	72 (2)	0.56 (0.42–0.74)	1.13 (0.80–1.61)
Asthma	219 (3)	114 (3)	105 (2)	0.77 (0.59-1.00)	187 (2)	0.85 (0.70–1.04)	105 (3)	0.92 (0.71–1.20)	82 (2)	0.78 (0.59–1.04)	0.65 (0.49–0.87)
Status asthmaticus	12 (0)	<5	8 (0)		6 (0)	0.50 (0.19–1.33)	<5		<5		
Bronchiectasis	12 (0)	8 (0)	<5		<5		<5		<5		

^a^: ID vs. gPop; ^b^: Within the cohort. Note: Relative risks (RRs) are given with 95% confidence intervals (CIs). RRs are presented only if both groups to be compared are comprised of at least five individuals.

**Table 4:** Prescription of drugs for obstructive airway diseases stratified by diagnoses of asthma and chronic obstructive airway disease in a cohort of people with intellectual disabilities (ID) and a referent sample from the general population (gPop).

Drugs	gPop				ID			
	No Diagnosis (*n* = 7538)	Asthma Only (*n* = 179, 45% ^a^)	COPD Only (*n* = 165, 41% ^a^)	Asthma and COPD (*n* = 54, 14% ^a^)	No Diagnosis (*n* = 7660)	Asthma Only (*n* = 89, 32% ^a^)	COPD Only (*n* = 151, 55% ^a^)	Asthma and COPD (*n* = 36, 13% ^a^)
	*n* (%)	*n* (%)	*n* (%)	*n* (%)	*n* (%)	*n* (%)	*n* (%)	*n* (%)
At least one R03	1122 (15)	156 (87)	160 (97)	54 (100)	698 (9)	73 (82)	142 (94)	35 (97)
R03A: Adrenergics, inhalants	932 (12)	135 (75)	157 (95)	54 (100)	490 (6)	65 (73)	129 (85)	33 (92)
R03BA: Glucocorticoids	495 (7)	57 (32)	110 (67)	34 (63)	208 (3)	25 (28)	88 (58)	22 (61)
R03BB: Anticholinergics	141 (2)	112 (63)	20 (12)	39 (72)	73 (1)	52 (58)	18 (12)	16 (44)
R03C: Adrenergics for systemic use	21 (0)	1 (1)	5 (3)	0 (0)	194 (3)	11 (12)	52 (34)	12 (33)
R03D: Other systemic drugs	45 (1)	11 (6)	26 (16)	13 (24)	69 (1)	10 (11)	38 (25)	5 (14)

^a^: Percentage based on the number of people with at least one diagnosis.

**Table 5:** Relative risks (RRs) with 95% confidence intervals (CIs) for ID versus gPop stratified by sex and diagnoses of COPD and asthma.

Drug		Men	Women
COPD	At least one R03	0.97 (0.85–1.11)	0.95 (0.88–1.04)
	R03A: Adrenergics, inhalants	1.03 (0.87–1.21)	0.91 (0.78–1.05)
	R03BA: Glucocorticoids	1.09 (0.75–1.58)	0.83 (0.54–1.26)
	R03BB: Anticholinergics	0.81 (0.62–1.05)	0.88 (0.69–1.14)
	R03C: Adrenergics for systemic use ^a^		21.58 (2.86–163)
	R03D: Other systemic drugs	0.80 (0.36–1.74)	2.29 (0.81–6.47)
Asthma	At least one R03	0.99 (0.93–1.05)	0.95 (0.90–1.00)
	R03A: Adrenergics, inhalants	0.92 (0.83–1.01)	0.88 (0.82–0.95)
	R03BA: Glucocorticoids	0.88 (0.71–1.10)	0.91 (0.73–1.12)
	R03BB: Anticholinergics	1.06 (0.62–1.81)	0.45 (0.26–0.78)
	R03C: Adrenergics for systemic use	9.28 (3.40–25.4)	38.0 (5.30–272)
	R03D: Other systemic drugs	1.21 (0.68–2.16)	1.36 (0.80–2.29)

^a^: Not assessed for men due to low numbers of prescriptions.
